# Lignocellulose Biomass Liquefaction: Process and Applications Development as Polyurethane Foams

**DOI:** 10.3390/polym15030563

**Published:** 2023-01-21

**Authors:** Marius Gabriel Bontaş, Aurel Diacon, Ioan Călinescu, Edina Rusen

**Affiliations:** 1 Faculty of Chemical Engineering and Biotechnologies, University Politehnica Bucharest, Gh. Polizu Street, 011061 Bucharest, Romania; 2 S.C. Protect Chemical S.R.L., 6 Cercetătorilor Street, 042024 Bucharest, Romania; 3 Military Technical Academy “Ferdinand I”, 39-49 George Coșbuc Boulevard, 050141 Bucharest, Romania

**Keywords:** lignocellulose biomass, liquefaction, polyols, polyurethane foams, biodegradability

## Abstract

One of the main strategies for sustainable human society progress is the development of efficient strategies to limit waste production and maximize renewable resource utilization. In this context, this review highlights the opportunity to transform vegetable biomass residues into valuable commercial products. Biomass conversion entails the depolymerization of lignocellulosic biomass towards biopolyols and the synthesis and characterization of the valuable products obtained by using them. The influence of the reaction parameters in both acid and basic catalysis is highlighted, respectively the influence of microwaves on the liquefaction reaction versus conventional heating. Following the depolymerization reaction, polyols are employed to produce polyurethane foams. As a special characteristic, the addition of flame-retardant properties was emphasized. Another interesting topic is the biodegradability of these products, considering the negative consequences that waste accumulation has on the environment.

## 1. Introduction

**Figure 1:**
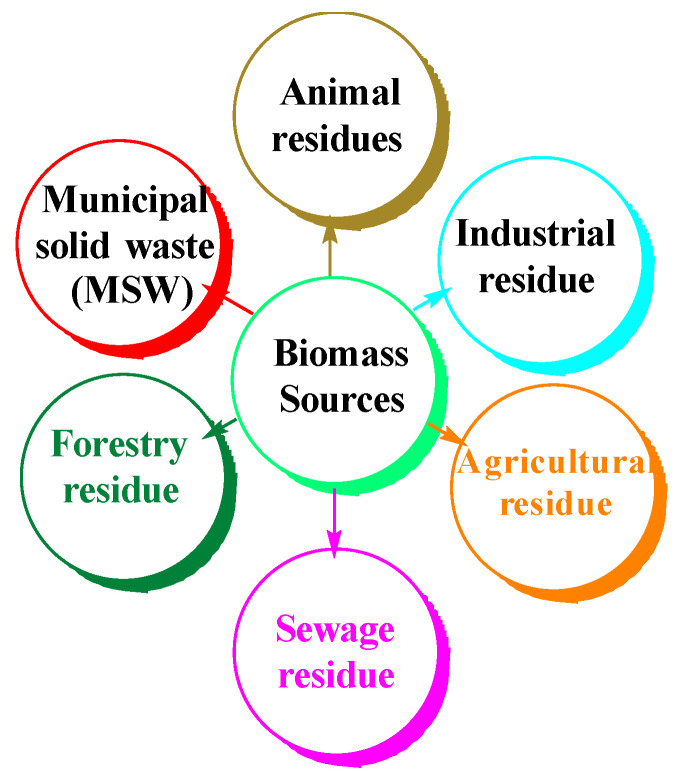
The most important biomass sources (adapted from [2]).

Biomass is at present the most widespread form of renewable energy, and its valorization is constantly increasing due to the concerns about the devastating impact of fossil fuel consumption, climate change, global warming, and their negative impact on human health [1]. The most important sources of biomass are presented in Figure 1 [2].

**Figure 2:**
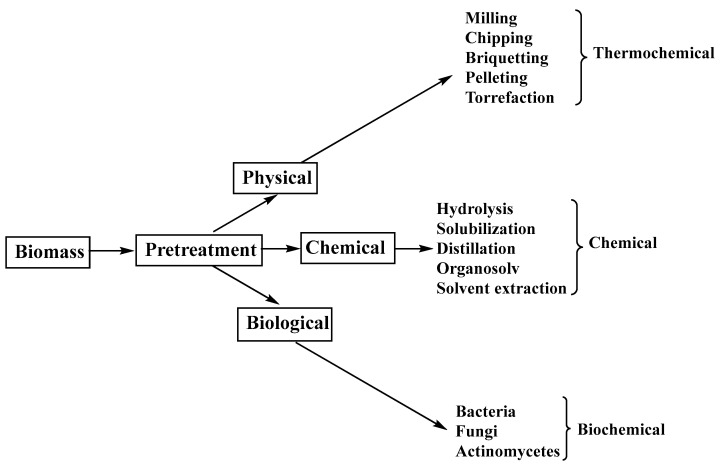
Biomass processing methods.

The most frequently used methods for biomass processing [3], presented in Figure 2, include mechanical, thermochemical, chemical, and biochemical processing.

The advantages of using biomass:

- It represents a renewable energy resource. When considering the valorization of the natural residues to generate energy, it can be considered practically inexhaustible due to the continuous waste generation in nature.
- The utilization can lead to the reduction of greenhouse gas emissions. The emissions during the burning process are reabsorbed during the formation process of the new biomass. However, the overall CO_2_ balance depends on the biomass processing strategy.
- It has a low market price. Compared to conventional fuels, biomass recovered energy has the potential to offer a more economical alternative, with a reduction of the costs up to 33%.
- Biomass constitutes an abundant global resource. Almost all regions on the planet can permit the generation of natural biomass waste, which is available for local use. In addition, generally, there is no need for large infrastructure to make biomass available for use.

Although there are not many, the disadvantages of using biomass-derived energy must be considered:

- In some areas, due to local conditions, the recovery of biomass can be more expensive than in others. This is an important aspect when considering the biomass collection, pretreatment, and storage of different types of biomass.
- It requires large land areas for the processes utilized to obtain energy from biomass, especially for storage, because the residues tend to display a low energy density.
- In some cases, the overutilization of this energy resource can cause damage to ecosystems or fragmentation due to the activities during the biomass residue collection process.

Biomass residues are considered alternative sources of energy which can reduce or eliminate the dependence on fossil fuels [4]. The use of fossil carburants has a negative impact on the environment due to the emission produced and the accumulation of CO_2_ in the atmosphere which is a factor in the global warming process [5]. Society must migrate towards the utilization of renewable energy resources such as solar, wind, or biobased fuels [6]. The biomass type and its production process affect its usage and economic value. Literature highlights six important criteria for biomass selection: accessibility, conversion technology, production costs, process efficiency, cost of raw biomass, production costs, and amount of emission generated during its processing.

This review covers biomass conversion processes to value-added products by liquefaction using polyols or phenol and hydrothermal processes with a special interest in polyurethane-based final products. The influence of the most important parameters (temperature, catalyst, reaction time, etc.) on the above-mentioned conversion processes is analyzed. The second part of the paper is focused on the synthesis of polyurethane materials using the polyols resulting from biomass valorization.

## 2. Polyols

**Scheme 1:**
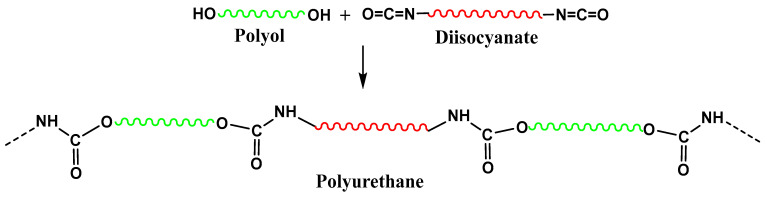
Polymerization between polyol and diisocyanate to form PU.

The general terminology of polyols refers to molecules that have two or more hydroxyl functional groups. From the point of view of molecular weight (M_w_), they can be polyols or even polyols-polymers, which can be polyesters, polyethers, polycarbonates, or polyacrylates [7]. The polyols with a low molecular weight, such as ethyleneglycol or glycerol, are used as raw materials in organic chemistry processes such as esterifications [8], dehydrations [9,10], hydrogenations [11,12,13], as well as in the macromolecular chemistry, in the synthesis of alkyd resins as cross-linking agent [14] and as chain extender [15]. Polyols are extremely important in different practical applications. Thus, polyethers polyols are mainly used in the synthesis of polyurethane foams (PU). To achieve this, a polycondensation reaction between a diisocyanate and a polyol is used (as presented in Scheme 1).

Unfortunately, a large part of the polyols used for PU is industrially produced using petrochemical derivatives, which are depletable and this justifies an increased interest in biopolyols derived from renewable resources [16,17]. For this reason, this review aims to present the newest, respectively, the most important methods to produce biopolyols from renewable resources, one of the most important raw materials being the lignocellulose biomass [18,19].

**Figure 3:**
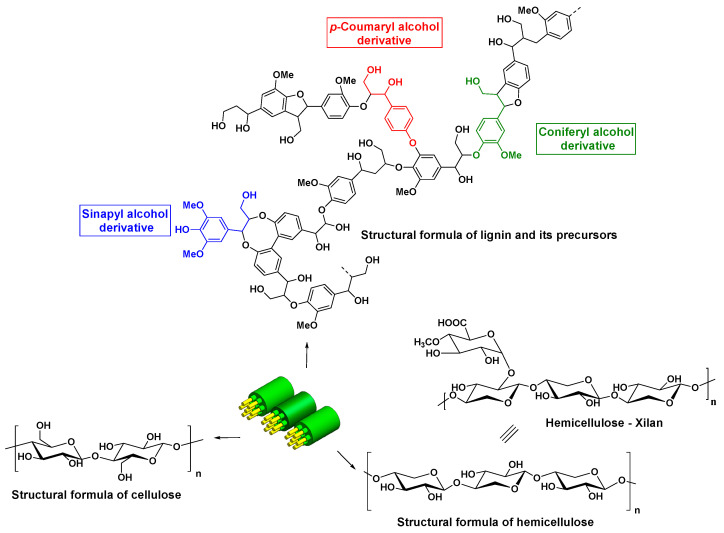
Structuring of lignocellulose biomass and structural formula of the components (Adapted from [20]).

The main structural units of lignocellulosic biomass are cellulose (30–35%), hemicellulose (15–35%), and lignin (20–35%) [20] all of which are highly functionalized materials rich in hydroxyl groups, making them promising feedstocks to produce bio-based polyols (Figure 3). In this context, because the biomass is solid, it must be brought to a liquid state for further reactions to be facilitated.

**Scheme 2:**
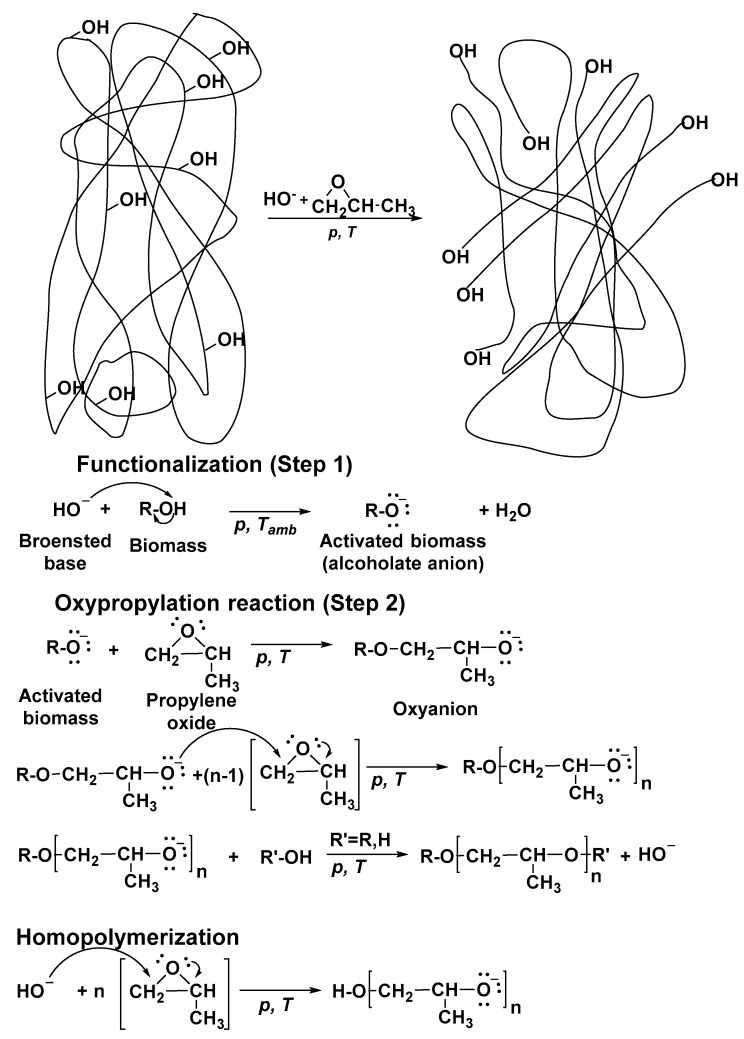
The mechanism of the oxypropylation reaction (Adapted from [26]).

The lignocellulose biomass liquefaction can be achieved by two routes: oxypropylation [21] and solvolysis (the actual liquefaction) [22,23,24]. The first method consists of the grafting of propylene oxide oligomers on the cellulosic main polymer chain [25,26]. The inconvenience of this reaction is the requirement of high pressure and a rather high temperature (100–200 °C), but the products obtained consisting of grafted cellulose, propylene oxide, and unmodified biomass form a mixture that can be used directly to obtain PU foams [26]. Presented in Scheme 2 is the general oxypropylation reaction, respectively, the mechanism of this reaction [26].

The second method entails atmospheric pressure and temperatures of 150–250 °C, using both basic and acidic catalysts. As solvents, glycerol and ethylene glycol are frequently used [27,28,29,30,31]. 

## 3. The Mechanism for Liquefaction of Lignocellulosic Biomass in the Presence of Polyols

**Scheme 3:**
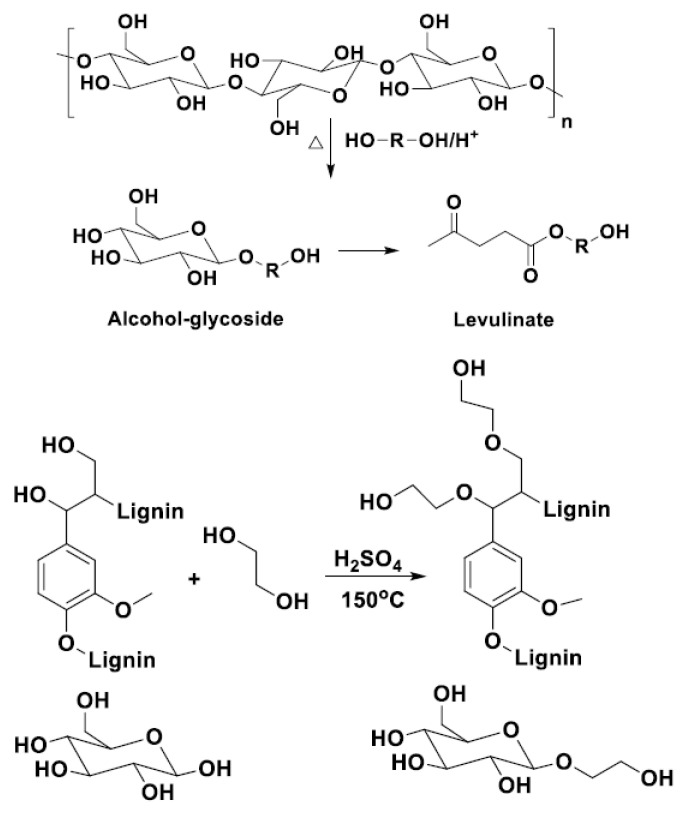
Lignocellulose solvolysis.

The lignocellulose biomass liquefaction in the presence of polyols involves the breaking of glycosidic chemical bonds, resulting in the synthesis of polyols and fragments of biomass [32,33]. Usually, lignocellulose, hemicellulose, and amorphous cellulose liquefaction are performed in the first stages of the process, due to more facile access of the solvent between the polymeric chains. In the case of crystalline cellulose, the process takes place at a later stage [34,35], and being one of the slower stages, it determines the reaction rate [36,37]. The cellulose undergoes breakdown into glucose or smaller molecules which can react with the liquefaction agent forming glycoside derivates [32,33,36], which in the end can lead to the formation of levulinic acid or levulinates as presented in Scheme 3.

Unfortunately, during the liquefaction process, undesirable recondensation reactions also take place [31,38,39]. The next topic of this mini review will focus on the analysis of the reaction parameters (type of biomass, type of catalyst, solvent, time, and temperature) on the liquefaction process.

## 4. Influence of the Reaction Parameters on the Lignocellulose Solvolysis Process

### 4.1. Type of Lignocellulose Biomass

**Scheme 4:**
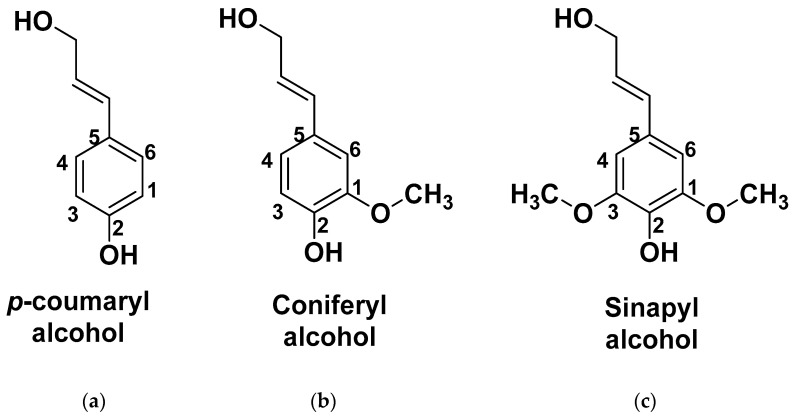
Main units of lignin (**a**) *p*-coumaryl alcohol; (**b**) Coniferyl alcohol and (**c**) Sinapyl alcohol.

Comparing the lignocellulose biomass from hardwood with softwood type, it is noticed that the latter undergoes a faster liquefaction process; however, the recondensation reactions are more frequent [35]. This can be explained by the existence of large amounts of guaiacyl propane (coniferyl alcohol) units in the softwood that are more reactive than the syringyl propane (sinapyl alcohol) units from the hardwood [39] (Scheme 4).

As expected, the granulometric biomass distribution influences the solvolysis process, with an increased surface area leading to an increased reaction rate.

### 4.2. Liquefaction Solvent

The solvent used for the liquefaction process is a central parameter, determining the degree of depolymerization and the structure of the final products [40]. Thus, a higher solvent polarity leads to an increased liquefaction efficiency. Solvent mixtures, i.e., propylene glycol and glycerol in different ratios, are commonly used as depolymerization mediums [41,42]. Glycerol is often used because it can obstruct, to a certain degree, the recondensation reactions [27].

The liquefaction reaction medium ratio is extremely important. Usually, the reaction is performed using an excess of polyols between 3 and 5 to 1 (biomass), to avoid recondensation reactions. The liquefaction selection is conducted considering the final utilization of the obtained polyols and biomass residues. Thus, to obtain rigid and semirigid PU foams, a mixture of glycerol and PEG 400 was employed [28], while for weather-resistant, semirigid foams PEG 4000 is frequently used [43].

**Scheme 5:**
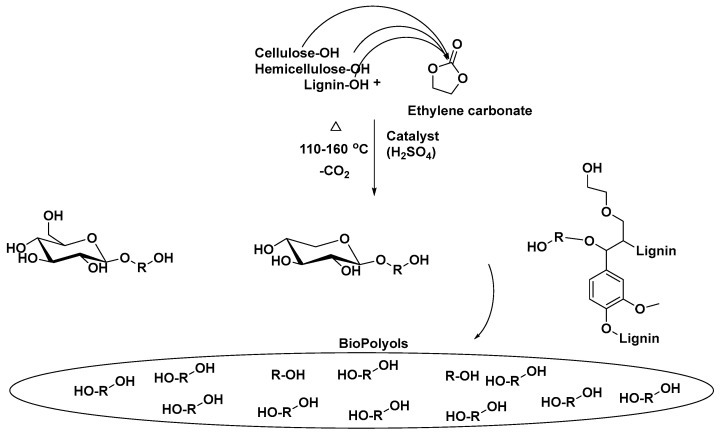
Ethylene carbonate glycolysis of lignocellulose biomass.

The most intensely used glycolysis agents are PEG, glycerol, ethylene glycol, and ethylene carbonate (EC), with the highest reaction rate being registered for EC [44], due to its high permittivity value [45,46,47] which makes it a very good choice for microwave-assisted glycolysis. The reaction steps in EC glycolysis are presented in Scheme 5.

**Figure 4:**
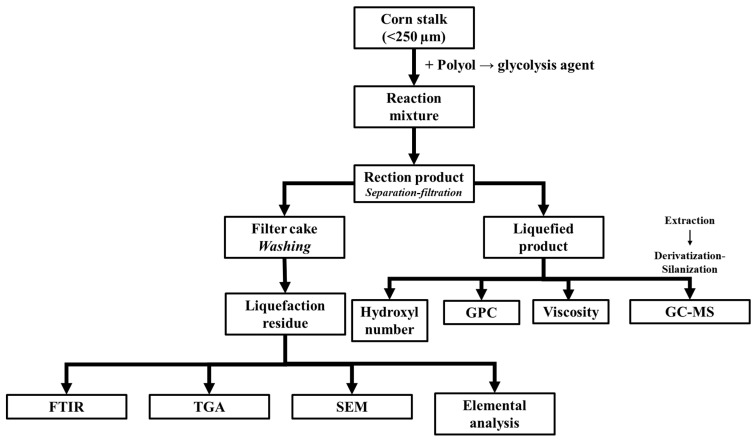
Flowchart for the research and development of lignocellulose glycolysis process (Adapted from [47]).

The flowchart for the research and development of a lignocellulose biomass glycolysis process is presented in Figure 4 [47].

The first stage of the technological process (Figure 4) consists of the grinding and mechanical separation of the fraction with a size of less than 250 µm from the corn stalks [47]. After this stage, the selected lignocellulose biomass reacted with polyols for the depolymerization reaction in basic or acidic conditions in accordance with the selected procedure. After the first reaction step, a solid/liquid heterogeneous reaction mixture will be still present. As a separation technique, filtration is used and the two components will be treated separately. The precipitate/filter cake represents the residue of the liquefaction reaction and can serve as a filler for composites, this is the reason for the use of different investigation procedures: FTIR (functional groups present determination), SEM (morphology assessment), TGA analysis (thermal resistance behavior) and elemental analysis (the overall elemental composition). The filtrate is separately characterized by hydroxyl number (the content of free OH groups), GPC (average molecular weight), GC-MS, and viscosity. Thus, the functionality (number of OH groups), molecular weight, and viscosity are critical parameters that influence the properties of the products obtained using the polyols, i.e., the polyurethane foams [47].

### 4.3. Catalysts, Temperature, and Reaction Time

The liquefaction reaction can take place both in acid [34,42,48] and alkaline [49,50] catalysis, but the latter received less attention in the literature. The most intensively used acid catalysts are sulfuric acid and phosphoric acid, or in some cases a mix of the two, while usually the optimal concentration is around 4% (weight %) in the reaction mixture. A higher catalyst concentration leads to the formation of recondensation products.

Based on the literature [51], sulfuric acid is the most utilized catalyst. Lu et al. [52] highlighted that the lignocellulose biomass depolymerization reaches a higher conversion in the case of alkaline catalysis using NaOH.

An advantage of alkaline catalysis would consist in lower corrosion of the metallic parts of the installation; however, the liquefaction reaction performed by this route is less studied. Nevertheless, the influence of the reaction parameters on the liquefaction of wheat straws using alkaline catalysis (NaOH) and crude glycerol was recently presented by Chang et al. [53]. Their results confirmed that an increase of the temperature from 180 to 240 °C, at 7.5% biomass loading, 3% NaOH, and 3 h reaction time, leads to an increase of the conversion from 50 to almost 80%, while the viscosity and hydroxyl number both decreased. Increasing the catalyst concentration afforded an increased biomass conversion with a decrease of the viscosity and hydroxyl number; however, the latter stabilized for a catalyst concentration of over 4% (weight %), 7.5% biomass content at a reaction temperature of 220 °C and 3 h reaction time.

The influences of various reaction parameters on the liquefaction of wood meal (birch) in two types of polyethylene glycol (400 and 200) and glycerol, the presence of a basic catalyst (NaOH) have been investigated by Maldas and Shiraishi [49]. As expected, the increase in the reaction temperature leads to a decrease in the residue; however, with the increase in the reaction time the polycondensation side reactions are favored, which determines the existence of a threshold beyond which the amount of residue begins to increase, a behavior that is observed even at a high polyol to biomass ratio of 5.5 (mass ratio). The optimum liquefaction conditions of wood meal in polyol PEG400 were found as temperature 250 °C; time 1 h; NaOH concentration 5% and polyol: wood, 4:6 (wt. ratio).

Hassan and Shukry [42] studied the influence of the reaction parameters on the acidic catalyzed liquefaction process of cotton stalks, respectively bagasse, using sulfuric acid as a catalyst. A biomass-to-polyol ratio of 1/4 was preferred in this study. PEG400 alone is not recommended as a liquefaction solvent as the maximum liquefaction yield did not reach 90% even at a longer liquefaction time. Replacing 10% from PEG400 with glycerol decreased the residue content remarkably from 18.4 to 6.9% and from 21.3 to 12.1% for bagasse and cotton stalks, respectively. Increasing the acid concentration up to 5% improved the liquefaction yield while the minimum liquefaction temperature was 150 °C, to provide very low residue content. There were no recondensation reactions in this system at a high acid concentration (5%), high liquefaction temperature (180 °C), or even at a longer liquefaction time (105 min).

The liquefaction temperature depends on the type of catalyst employed, and thus in the case of acid catalysis, the temperature range is between 15 and 70 °C, while for alkaline catalysis higher temperatures are required reaching up to 250 °C. The reaction time depends on the temperature at which the process is conducted, and the type of catalyst employed, nevertheless, it needs to be limited up to 4–6 h otherwise it will fail to be economically viable. The first stages (15–30 min) of the liquefaction are rapid, involving the attack on the lignocellulose, hemicellulose, and amorphous cellulose [54]. The reaction rate decreases after this interval, due to the limiting stage, which is the diffusion between the crystalline cellulose fibers and their glycolysis [54]. In acidic catalysis conversions of over 90% can be reached for a reaction interval of 90 min at 150 °C, while for alkaline catalysis this would require a significant temperature (of around 100 °C).

The reaction time is a very important parameter. Usually, the lignocellulose biomass requires a depolymerization reaction time that is between 5 and 40 min, in the case of conventional heating processes [55,56]. The reaction time can be reduced using process intensification strategies such as microwave heating [57,58]. 

### 4.4. Heating Method—Process Intensification Strategies 

Microwave (MW) heating can offer a more rapid and efficient alternative to conventional heating methods which are relatively slow and inefficient due to low thermal conductivity in the entire volume. In the case of conventional heating, the surface of the material is initially heated, and then the heat is transferred to the inside of the material. This means that there is a certain temperature gradient in the material. The application of microwave heating is an alternative way to achieve faster and more homogeneous heating of the entire sample volume due to a higher penetration of the microwaves inside the reaction mixture [59]. Microwave irradiation produces efficient internal heating when direct coupling of microwave energy with the molecules of solvents, reagents, or catalysts is achieved. In addition, MW heating can offer a route for rapid chemical processes due to the thermal effect, i.e., the high process temperature obtained by the microwave heating of polar materials which leads to a higher rate of chemical reaction following the Arrhenius law. In addition, during microwave heating, there are also specific thermal effects caused by accelerations of chemical transformations in the magnetic field that cannot be achieved or reproduced in conventional heating [59,60,61]. Microwave heating has many advantages, for example, low energy use, very high heating rate, improved treatment time, better physical and chemical properties (due to shorter exposure to higher temperatures), simplicity, and lower risk for the environment. These attributes assigned to microwave heating have not been observed during heating with conventional methods [62,63,64]. 

Li et al. [65] studied the microwave liquefaction kinetics of corn stover in the presence of ethylene glycol (EG) using sulfuric acid as a catalyst. The apparent reaction rate constant (k) of the liquefaction was examined using a first-order reaction model. The k values of corn stover increased from 0.080 min^−1^ to 0.165 min^−1^, for a reaction temperature ranging from 120 °C to 180 °C. The k value for cellulose liquefaction at 160 °C was close to that of corn stover, which sustains that the rate-determining step in microwave liquefaction is cellulose glycolysis [65]. The microwave liquefaction rate of corn stover at 160 °C was seven times greater than that of conventional heating liquefaction [65]. This was due to the decrease in apparent activation energy and the increase in the frequency factor as compared to conventional liquefaction kinetic parameters which indicate a non-thermal effect of microwave in the liquefaction of corn stover, which can explain the acceleration mechanism of microwave-assisted liquefaction [65].

**Figure 5:**
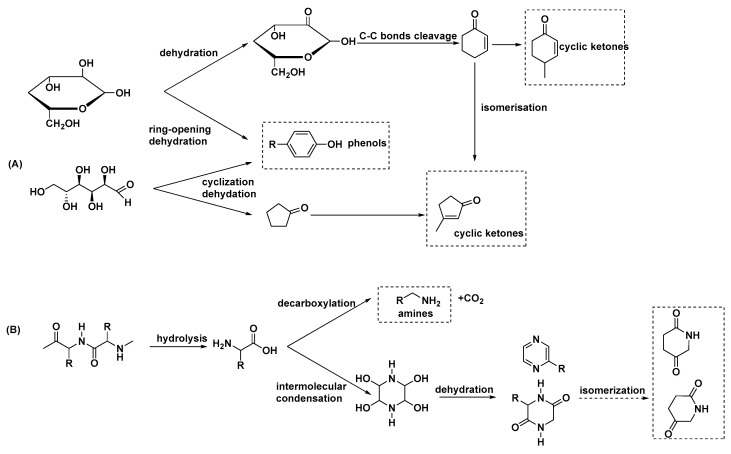
Plausible pathways for bio-oils formation via hydrothermal liquefaction (Adapted from Kruse et al. [69]).

Hydrothermal liquefaction (HTL) uses feedstocks with a high humidity content to produce biofuels and other chemical products [66]. The processing conditions in the case of HTL include a temperature range of 250–375 °C and a pressure domain of 4–25 MPa [67,68]. The presence of water facilitates the hydrolysis, degradation, and repolymerization of cellulose, hemicellulose, and lignin, which are converted into biofuels, such as bio-oil, char, and gases [69]. The first reaction consists of the hydrolysis of the biomass into polysaccharides, oligosaccharides, and monosaccharides, followed by the formation of phenols, cyclic ketones, and after volatiles and char formation as a result of the decarboxylation, dehydration, and fragmentation reactions (Figure 5).

**Figure 6:**
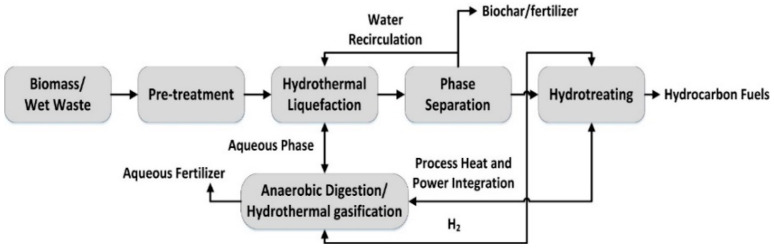
Process flow diagram of HTL process of lignocellulose biomass (Adapted from Gollakota et al. [67]).

In the case of lignin, hydrolysis, and decomposition take place, a process flow diagram is presented in Figure 6 [67]. 

Water is usually used as a solvent for HTL, but relatively recent studies [70] present the utilization of different solvents with the obtaining of methanol and ethanol as final products. The catalysts play a significant role in the mechanism of the reaction. Thus, a higher yield of bio-oil was observed using alkali catalysts by Minova et al. [71], which can be explained by the inhibition of char formation due to the catalyst characteristics.

**Scheme 6:**
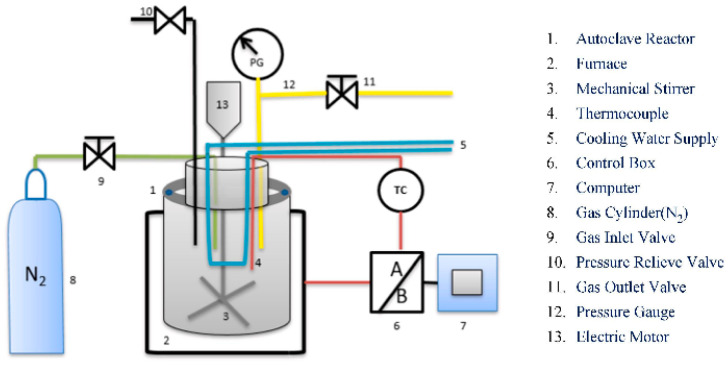
Schematic diagram of high temperature, high-pressure autoclave reactor (Adapted from [66]).

A recent study by Mathanker et al. [66], presents the HTL of lignocellulose in an installation presented in Scheme 6. The authors determined the influence of the reaction temperature, catalyst, and other operating parameters on the liquefaction process.

Presented in Table 1. Product distribution at different conditions of temperature, pressure, and retention time (Adapted from [66]). Table 1 is the product distribution depending on the temperature, pressure, and residence time for the hydrothermal liquefaction process of corn stover lignocellulose biomass. The highest conversion into heavy oil was 29.25% at a temperature of 300 °C and a pressure of 2000 psi [66]. The GC-MS results indicated that the heavy-oil fraction is made up mostly of phenolic compounds [66]. The gas obtained consists of a mixture of CO_2_, CH_4_, C_2_H_4_, C_2_H_6_, C_3_H_6_, C_4_H_8_, C_4_H_6_, C_5_H_12_, C_6_H_12_ and C_6_H_14_ [66].

Where, HC: Hydrochar, WSH: Water soluble hydrocarbons, 250│15: set temperature 250 °C and retention time 15 min, 300/1100: initial pressure: 300 psi and final pressure 1100 psi.

(1) $$Hydrochar\;wt.\;\%=\frac{W_{HC}}{W_{CS}}\times 100$$

(2) $$Conversion\;wt.\;\%=\frac{W_{CS}-W_{HC}}{W_{CS}}\times 100$$

(3) $$Aqueous\;oil\;wt.\;\%=\frac{W_{AO}}{W_{CS}}\times 100$$

(4) $$Heavy\;wt.\;\%=\frac{W_{HO}}{W_{CS}}\times 100$$

(5) $$Gas\;wt.\;\%=\frac{W_{F}-W_{R}}{W_{F}}\times 100$$

(6) $$WSH\;wt.\;\%=100-\left(W_{HC}+W_{AO}+W_{HO}+W_{Gas}\right)$$

(7) $$W_{F}=Weight\;of\;feed\;\left(corn\;stover+deionized\;water\right)$$

(8) $$W_{R}=Weight\;recovered\;from\;reactor$$

The high energy costs of the hydrothermal processes determine the development of novel liquefaction methods at lower temperatures between 12 and 50 °C and low pressure or even atmospheric pressure in acidic catalysis. The latter is termed moderate acid-catalyzed liquefaction (MACL) and it employs as solvents polyols or phenols [7].

### 4.5. Mechanism of Acid-Catalyzed Liquefaction of Cellulose and Lignin in Phenols

**Figure 7:**
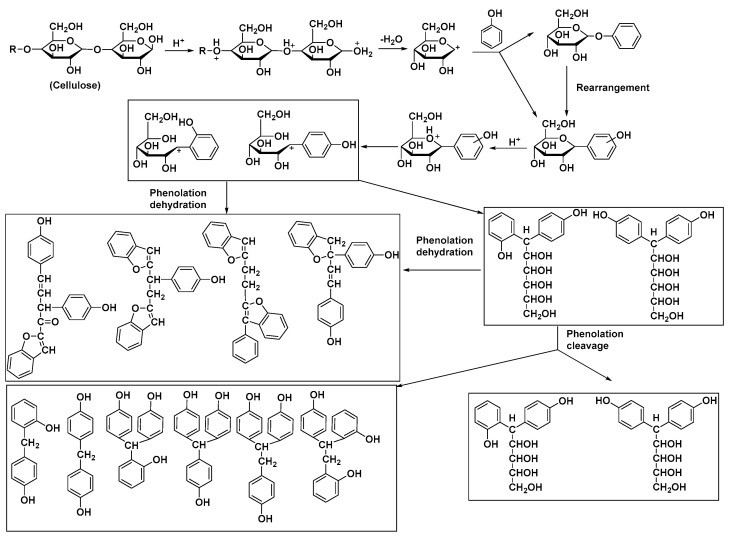
A proposed liquefaction pathway for cellulose in the presence of phenol under acid-catalyzed conditions (Adapted from [72]).

The first stage of acid-catalyzed liquefaction of cellulose and lignin in phenols involves the breaking of the glycosidic bonds and the formation of phenyl glucopyranosides [72] (Figure 7). The reaction products are rich in phenol derivatives, which in the presence of formaldehyde afford adhesives [73].

**Figure 8:**
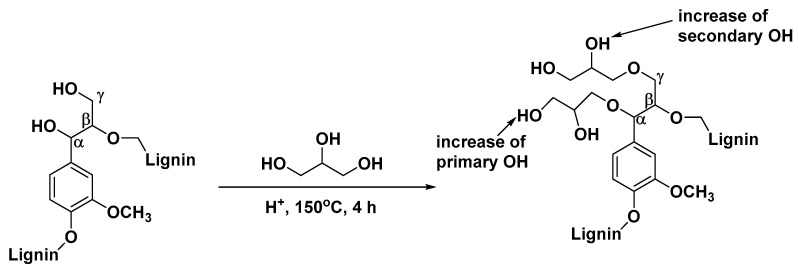
Reaction between lignin and glycerol—acid-catalyzed (p-toluene sulfonic acid) wood liquefaction (Adapted from [74]).

The liquefaction in the presence of polyols in an acid medium leads to the introduction of hydroxyl groups on the structure of lignin, which increases the solubility of the products in the reaction medium. The total solubilization is accomplished by complete degradation due to temperature and the acid catalysts, the process leading to the formation of carboxylic aromatic acids and phenolics, which by subsequent hydrolysis afford derivatives of syringyl and guaiacyl (Figure 8) [74].

The introduction of ultrasounds in a system can have mechanical, chemical, and thermal effects caused by the formation and collapse implosion of microcavities. These microbubbles undergo growth up to a critical dimension after which they implode, generating in the process short-lived localized temperatures of 5000 K and pressure up to 1800 atm and with the possibility to generate free radical species [75]. These characteristics are a good explanation for increased biomass fragmentation using ultrasounds [76,77]. 

In a recent study, Mateus et al. [78] presented the influence of ultrasounds, respectively the acid catalysts (*p*-toluene sulfonic acid) on the liquefaction of lignocellulose biomass (cork residue).

Presented in Table 2 are the reaction rate constants in the absence and the presence of ultrasounds at various amplitudes, respectively. The results confirmed the introduction of ultrasounds led to a 4.5 increase in the reaction constant. 

### 4.6. Polycondensation Reaction in Lignocellulosic Liquefaction

The increase in liquefaction time leads to a wider range of product distribution, but the liquefaction effectiveness cannot be improved significantly until the reaction time reaches an appropriate value (a threshold point). The residue content would further increase if the reaction time is prolonged, while other factors would remain constant. This residue content rising as a function of time is explained by a polycondensation process, while it is referred to as a decomposition process before this stage. Therefore, there are mainly two reactions in the entire lignocellulosic liquefaction, i.e., decomposition and polycondensation.

Polycondensation reactions can be estimated from the relationship between residue content and liquefaction time [79]. If the residue content registers an increase during the liquefaction reaction after a certain period, the occurrence of polycondensation reactions is confirmed. In contrast, steadily decreasing curves (of the residue content) during the entire liquefaction process indicated that no polycondensation took place in the liquefaction system.

### 4.7. Polyols Obtained by Liquefaction

The most important application of the products obtained by liquefaction is the production of polyurethane foams. As previously presented, the polyurethane foams are the result between a diisocyanate unit and a polyol unit; therefore, the first limitation of the reaction is the physical compatibility between the two components. Considering this aspect, the liquefaction reaction, which entails the depolymerization of the biobased polymeric units, must be conducted to maximize the content of low molecular weight polymer units in the obtained polyols, which signifies a high-efficiency process. As the efficiency increases, the molecular weight of the product is lower, and its solubility (compatibility) with the diisocyanate is higher. The value of the hydroxyl index decreases during depolymerization, while the acidity index increases. The first is situated in the 100–600 mg KOH/g, while the acidity index varies between 0 and 40 mg KOH/g, the viscosity is up to 40 Pa∙s and the average molecular weight of the polyols is between 200 and 7000 g/mol.

## 5. Applications of Products Obtained by Biomass Liquefaction 

First of all, liquefaction reactions allow access to biobased value-added products such as biofuels and biochemical biomaterials [80]. One of the most important applications consists in the synthesis of biopolyols and their utilization for polyurethane products fabrication, such as polyurethane foams with high thermal and compression resistance, also presenting biodegradability [81]. In addition to foams, membranes for gas separation (CO_2_/CH_4_) were also obtained [82]. As expected, wood adhesives represent a direct application of bio-based polyurethanes [22]. Following a hydrothermal liquefaction process, biofuels with limited emission of SO_x_ and NO_x_ are also obtained [83].

Polyurethanes find applications in a wide variety of products, such as mattresses in furniture (flexible); cushioning, bumpers, sound insulation in automotive, components (flexible, semi-flexible, rigid PUFs); thermal insulation in refrigerators, refrigerated transport, cold storage and piping (rigid PUFs); soles in shoes (PU elastomers and elastomeric foams); and adhesives, coatings, and sealants (thermoset/thermoplastic PUs) [84,85]. The development of biobased products with similar or enhanced properties also presenting biodegradability can therefore bring significant economical, societal, and environmental benefits.

Depending on their physical properties, polyurethane foams can be classified into two major groups: flexible and rigid polyurethane foams. The Young modulus and the yield strength are very different for these types of polyurethane. These materials possess good thermal parameters being applicable over a wide range of temperatures (from −200 °C to +135 °C). The average thermal conductivity coefficient of polyurethane foams is 0.026 W/m^2^, and the most favorable apparent density after the curing of rigid foam is usually 35–50 kg/m^3^.

Flexible and rigid foams are low-density foams and semi-rigid foams have medium density [86]. The flexibility depends on the chemical composition as well as matrix polymer characteristics such as the degree of crystallinity and the degree of crosslinking. Polyurethane foams are the main type of cellular plastic. A wide range of materials such as soft flexible foams and tough rigid foams can be synthesized thanks to the versatile chemical characteristics of polyurethane chemistry [87,88].

**Figure 9:**
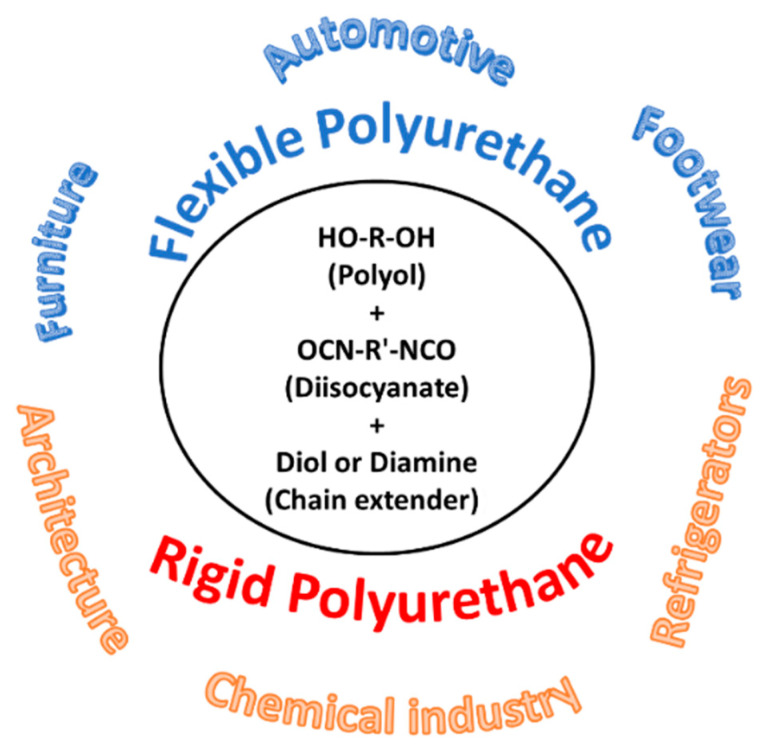
Applications of polyurethane foams (Adapted from [89]).

The polyurethane foams derived from the lignocellulose biomass liquefaction are used to obtain rigid and semirigid foams [89]. In the case of acidic catalysis, the polyols are initially neutralized with a base NaOH or MgO. Because the glycolysis process is usually conducted down to low molecular weight polyols, the resulting foams do not display flexibility, which leads to the formation of rigid foams or the need to add commercial polyols to obtain semirigid foams. The applications of polyurethane foams are presented in Figure 9.

Examples of rigid foams were obtained by P. Kosmela et al. [90] via partial substitution of petrochemical-derived polyols with bio-based ones, which were characterized by slightly increased apparent density and average cell size compared to unmodified materials. The best mechanical performance was observed for the sample containing 35% (weight %) of biopolyol in the polyol mixture, which indicates a synergistic effect between the applied polyols. The applied modification delayed the thermal degradation of foams due to changes in the thermal decomposition process.

The use of bio-based polyol for polyurethane foam also leads to an increase in the average cell size [90]. This aspect can be explained by the higher water content of the biopolyol (0.63 wt%) compared to the non-renewable biopolyol (<0.1 wt%) used in the study [90], which leads to a slightly higher generation of carbon dioxide during the reaction with isocyanate. In addition, the lower viscosity of the bio-polyol-petrochemical polyol mixture (7600 compared to 9200 mPa∙s) can be a contributing factor that facilitates the diffusion of CO_2_ and hydrofluorocarbon used [90]. Similar results related to the increase of cell size during the incorporation of bio-based polyols into polyurethane foams have been reported by others [87,91,92]. 

Previous studies confirmed that the viscosity of the biopolyols is an important parameter when considering the use of polyurethane foams [93] besides the molecular weight and the hydroxyl number [93]. 

The mechanical properties of polyurethane determine their use. For example, the compression resistance depends on the porosity and the interconnectivity of the network (the solid that forms the foam cell membrane). A high content of closed cells determines a higher compression resistance of the polyurethane foam [93]. 

A recent study [94] presents the synthesis of polyurethanes that contain 90% polyol derived from lignocellulose degradation. Polyurethanes are known as thermally stable materials due to the urethane linkage in their structure. The breaking down of the polyurethane bond involves three stages: the formation of a diisocyanate and alcohol (240–310 °C), a primary amine, an olefin (320–470 °C), and a secondary amine (over 470 °C). 

The glass transition temperature (Tg) of the synthesized polyurethanes was determined by DMA analysis. From the data presented in Table 3, it can be observed that the possibility of using the materials for both indoor and outdoor applications. 

As expected, the liquefaction reaction time has an influence on the mechanical properties of the resulting foams through the molecular weight of the obtained polyols. Recently, an intense variation of the polyol molecular weights with the liquefaction reaction time was observed [95]. The molecular weights of the polyols are dependent on the reaction parameters (time) (Table 4).

From an economic point of view, the focus is directed toward the reduction of energy requirements and costs, which determines the utilization of the product directly after the liquefaction reaction without de removal of the residual product [96]. The presence of residue leads to the higher thermal stability of the polyurethanes, resulting in the increase of both the initial and maximum degradation temperature, but it also has a negative effect on the formation of urethane bonds.

**Figure 10:**
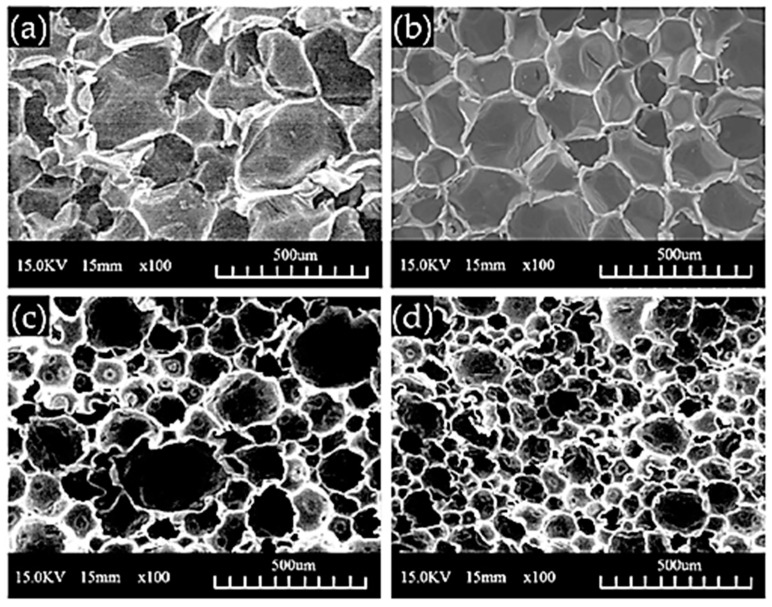
SEM images of polyurethane foam samples were prepared with reaction times of (**a**) 15; (**b**) 90; (**c**) 180; and (**d**) 300 min [88].

As previously presented, the viscosity of the polyols influences the nature of the pores of the polyurethane foams. The average diameter of the pores decreased from 350 to 100 µm (Figure 10) with the increase in the liquefaction reaction time [88], respectively with the decrease in the viscosity.

**Figure 11:**
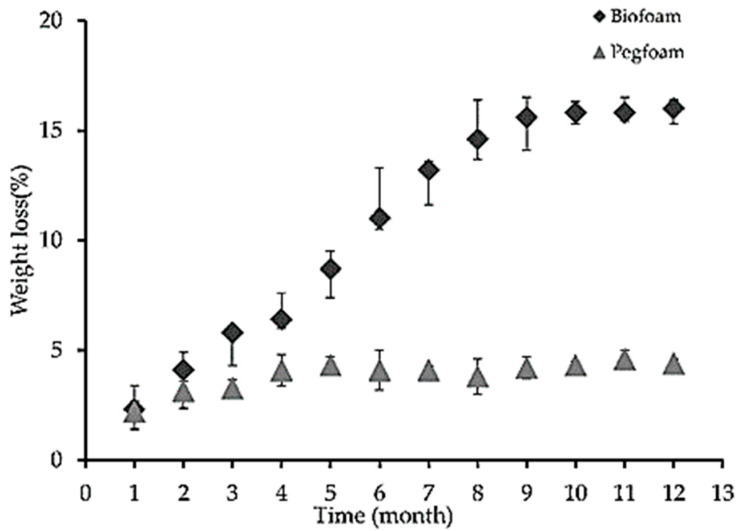
Weight loss of polyurethane samples over time in biodegradation experiments [88].

What is specific for this type of polyurethane foam is the biodegradability, measured as a weight-loss of the foam samples buried in the soil. Thus, a weight loss of around 17% was observed for 12 months (Figure 11).

Rigid polyurethane foams are obtained by using biopolyols obtained by the liquefaction of wood meal (southern pine) [97] in the presence of PEG400 and glycerol. This depolymerization was performed in acidic catalysis (H_2_SO_4_) using microwaves [97]. Before the synthesis of the foams, the polyols were neutralized with a NaOH solution, followed by homogenization with catalyst, surfactants, water, and lastly with methylene diphenyl diisocyanate (MDI). The foams formulations employed are presented in Table 5.

The mechanical properties of the polyurethane foams were assessed, and it was determined that a longer liquefaction reaction time leads to inferior properties, which can be explained by the decrease in the hydroxyl index value. 

**Figure 12:**
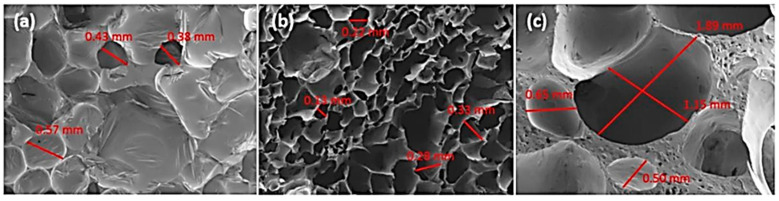
SEM images of (**a**) 80:20, (**b**) 60:40, and (**c**) 50:50 (weight ratio castor oil polyol: wheat straw derived biopolyol) and TDI foams [98].

**Figure 13:**
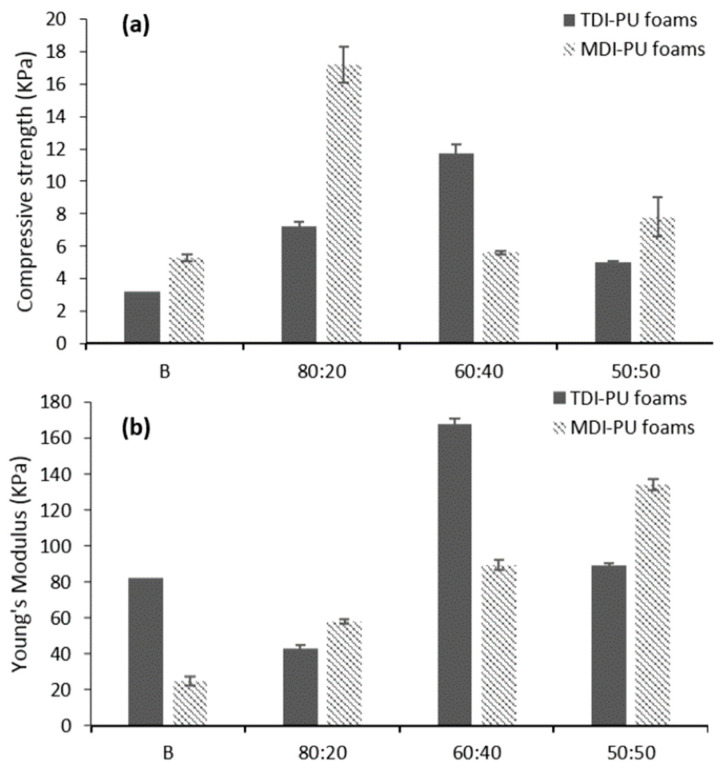
Mechanical properties of wheat straw derived biopolyol—formulated PU foams: (**a**) Compressive strength and (**b**) Young’s Modulus [98].

To reduce the amount of wheat straw residue, a liquefaction process using glycerol and H_2_SO_4_ was employed to produce polyols capable of replacing castor oil for the production of polyurethane foams [98]. The castor oil was replaced up to 50% (weight %) and as diisocyanate both MDI and toluene-2,4-diisocyanate (TDI) were used for a better comparison. Both the mechanical and thermal properties of the polyurethane foams were improved at a replacement value for castor oil of 40% [98]. At this optimum value, the obtained polyurethane foams display a compact and ordered morphology (Figure 12). In addition, the mechanical properties are dependent on the amount of biopolyol introduced with an optimum for the formulation containing 40% (Figure 13).

Additionally, the PU based on the 40% wheat straw-derived biopolyol presented an excellent biodegradability. At 30 days interval, the weight loss registered was 5.6 and 7.31% for the TDI and MDI formulations, respectively [98].

**Figure 14:**
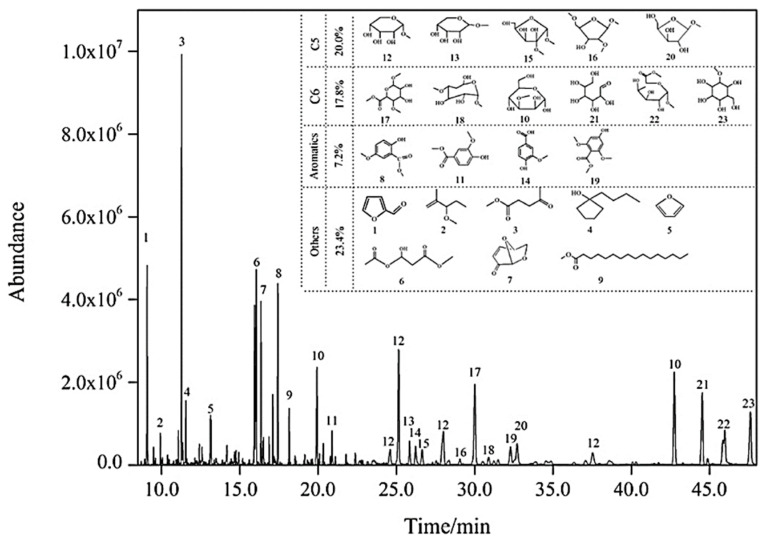
GC–MS chromatogram of bio-polyol via microwave liquefaction [99].

Polyurethane (foams) nanomaterials composites with cellulose nanocrystals (CNCs) as nanofillers are also presented in the literature [99]. The first step in their preparation was the liquefaction of rape straw under microwave irradiation using methanol and 3% H_2_SO_4_ as a catalyst. The nonrenewable commercial polyol was replaced up to 40% (PU40) and nanocellulose was added as filler 1 to 6% [99]. Utilizing GC-MS analysis, the composition of the polyols obtained under microwave irradiation was determined [99] (Figure 14). Both bio-polyol and cellulose nanocrystals were prepared from the liquid portion and solid residue of microwave-liquefied rape straw [99]. GC–MS, ^1^H NMR, and FTIR demonstrated that the bio-polyol is suitable to be employed for polyurethane foam due to the hydroxyl content. By replacing 20% of the non-renewable polyol with bio-polyol, the foam cell became more homogenous and finer, resulting in low thermal conductivity and high mechanical performance, while, further increasing bio-polyol content from 20 to 40%, the cell diameter was increased by 460% and both Young’s modulus and compressive stress decreased by 70%. However, the 40% bio-polyol foam could be remarkably reinforced by 4% CNCs because the hydroxyl-rich structure in CNCs increased the crosslinking density, resulting in the increase of physicomechanical performance of bio-foam [99].

**Figure 15:**
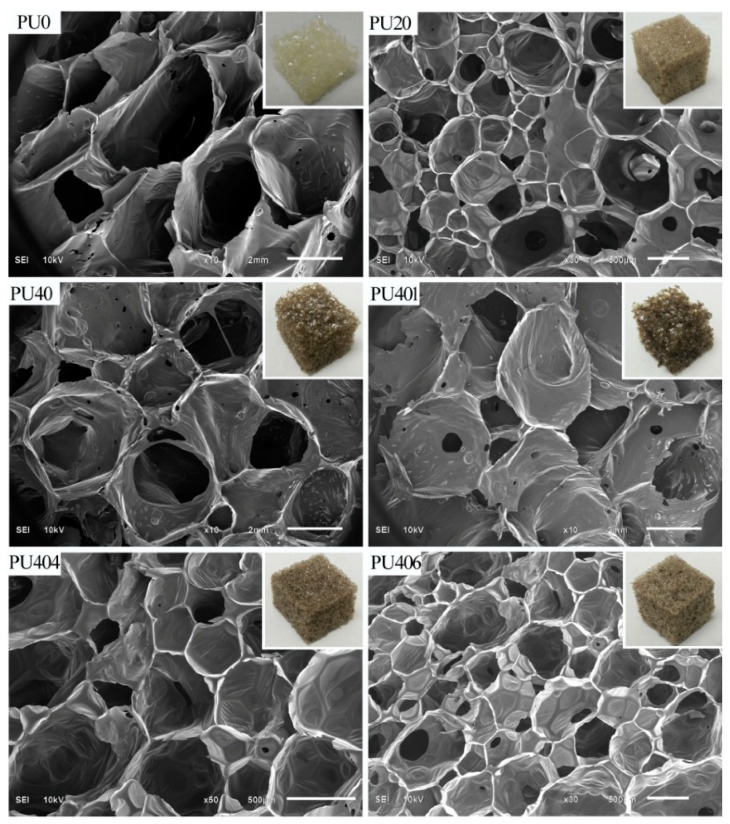
SEM images of bio-foams and cellulose nanocrystals reinforced bio-foams (Adapted from [99]).

The properties of the obtained polyurethane foams and nanocomposites are presented in Table 6 and their morphology in Figure 15 [99].

**Scheme 7:**
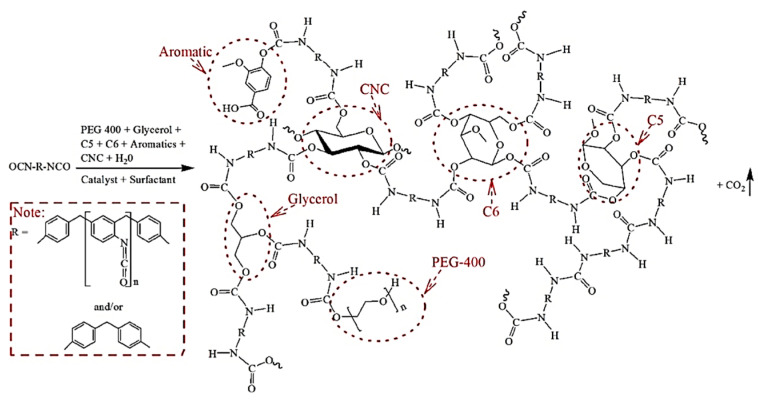
Proposed reaction scheme and possible chemical structure of CNC reinforced bio-foam (Adapted from [99]).

The possible reactions and chemical structure of the cellulose nanocrystals (CNC) reinforced bio-foams are presented in Scheme 7.

The reinforcement behavior of CNCs in the preparation of bio-polyol foams was evidenced by solid-state ^13^C NMR and FTIR analysis. As compared with the reference (PU40), Young’s modulus and compressive stress in the optimal 4% CNCs reinforced bio-foam increased by 590% and 150%, respectively [99]. 

**Figure 16:**
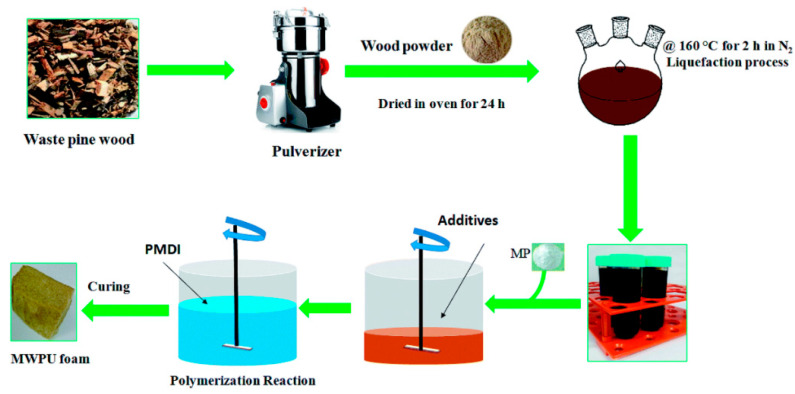
Schematic showing waste pine wood liquefaction and synthesis of melamine phosphate polyurethane foam.

A special application consists of the synthesis of flame-retardant polyurethane foams [100]. The stages of bio-foam fabrication are presented in Figure 16 and the flame-retardant additive is melamine phosphate (MP).

**Figure 17:**
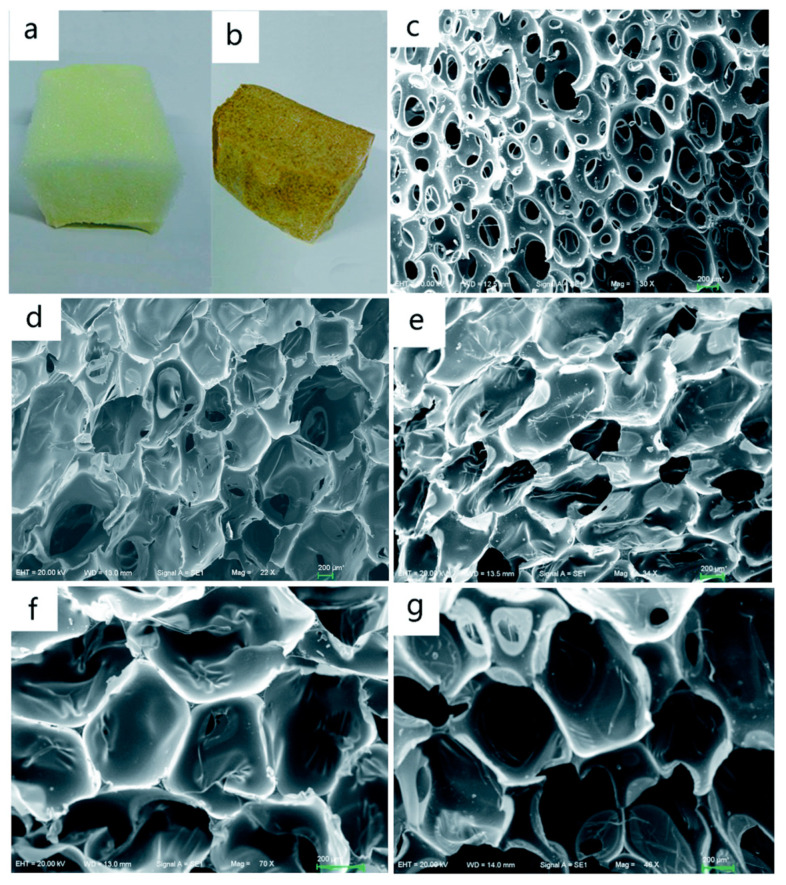
The digital photograph of the synthesized polyurethane foam (**a**) and MWPU foam (**b**); SEM photographs of the surface of the PU foam (P6) (**c**) and pine wood-based MWPU foams with different content of MP fillers: 0 wt% (**d**), 5 wt% (**e**) 7.5 wt% (**f**), 10 wt% (**g**) [100].

The optimum liquefaction temperature was 160 °C which resulted in aliphatic polyols with a higher reactivity than the aromatic polyols towards the diisocyanate and a urethane bond displaying greater thermal resistance [100]. The T_g_ of the foam containing 10% filler was 43.8 °C which is significantly higher than without the filler [100]. Moreover, the MP fillers improved the storage modulus and loss modulus of the resultant foams. Thus, the foams are greener and more environmentally friendly, which is expected to pave the way toward expanding their potential applications (Figure 17).

## 6. Discussion and Limitations

The HTL process has been developed to convert biomass into bio-oil/bio-crude fuels and organic derivatives. This method has limitations that make the scale-up difficult to be implemented due to the high reaction temperatures (200–400 °C) and pressure range (5–20 MPa). However, the quantity of residue is very small, almost inexistent and there is the possibility for straightforward obtained final products such as coatings and adhesives. HTL can be performed in the absence of a catalyst in terms of downstream processing, which constitutes an advantage, in addition to water serving as the solvent. MACL has the advantage of a lower cost due to a lower energy requirement, which can also be improved using ultrasounds and/or microwaves in the presence of an acidic catalyst. Nevertheless, the acidity of the medium requires a corrosion resistant installation both for the reaction and separation stage, since the solvolysis process doesn’t assure a complete conversion. However, MACL has the advantage of a lower energy requirement due to the use of a catalyst and the use of a solvent different from water can facilitate control over the properties of the final products. Both the nature of the employed solvent and catalyst influences the lignocellulose conversion process. In the case of MACL, often used as solvents for the production of biopolyols are glycerol, diethylene glycol, and polyethylene glycol oligomers, while the use of phenol as a solvent allows access to phenolic derivatives. If ethylene or propylene carbonate is used as a solvent, the release of toxic gas takes place. More recently, the literature presents the utilization of ionic liquids as a green solvent alternative; however, for the moment, the price prohibits large-scale implementation.

Comparing the processes, from the reaction products point of view, in the case of HTL bio-oil for energy applications is obtained. In contrast, MACL employing polyols permits the production of biopolyols for coating, adhesives, and foams, while the use of phenols as solvent leads to phenolic derivatives products suitable for applications in adhesive or active carbon fibers. A major drawback in the case of polyols-based solvolysis is the obtaining of reaction mixtures with a high molecular weight, and high viscosity for the reduction of which a higher quantity of solvent is required. In the case of MACL utilizing phenol, a high quantity of phenol is required, which has to be recycled.

Wood is still the largest biomass energy resource today. Other sources include food crops, grassy and woody plants, residues from agriculture or forestry, oil-rich algae, and the organic component of municipal and industrial wastes.

In conclusion, all lignocellulose biomass conversion methods presented have their respective advantages and limitations. However, through the optimization of the process, value-added products presenting the desired properties can be obtained.

## 7. Conclusions and Final Remarks

This review presents one of the most important problems at a global scale, the elimination or at least reduction of the dependency on oil-based raw materials through the synthesis of valuable products derived from biomass and ideally from residual vegetal biomass. In this context, the utilization of polyurethane foams in different domains is well established and their market demand continues to increase. The fabrication of the foams requires two components: polyols and diisocyanates.

The first part of this review covers the lignocellulose liquefaction process toward the production of biopolyols. Thus, the liquefaction process was presented in acidic and basic conditions with an emphasis on the reaction mechanism and the influence of the reaction parameters on the synthesized polyols.

The second part of the review was focused on the synthesis of polyurethane foams from polyols obtained by lignocellulose biomass liquefaction. The capacity of the bio-polyols to replace commercial/non-renewable polyols was highlighted with values of 50% coupled with the improvement of the mechanical and thermal properties of the foams. As expected, the liquefaction conditions, the hydroxyl index, and starting biomass characteristics are critical parameters that affect the nature and morphology of the obtained polyurethane foams.

Future studies must be directed towards bio-foams for special applications such as flame retardant. Nevertheless, the fields of application domains are much more extensive, making it an international challenge for research teams. Another important topic is the capacity of bio-foams to exhibit biodegradability. Probably, the new research in the field will be oriented towards increasingly higher degrees of biodegradation and diversification of the targeted applications of the bio-foams.

## Figures and Tables

**Table 1:** Product distribution at different conditions of temperature, pressure, and retention time (Adapted from [66]).

T (°C)│RT (min)	P_initial_/P_final_ (psi)	HC ^1^ wt.%	Conversion ^2^ wt.%	Oil Phase		Gas ^5^ wt.%	WSH ^6^ wt.%
				AO ^3^ wt.%	HO ^4^ wt.%		
250│15	300/1100	23.496	76.504	13.50	19.35	12.291	31.363
300│15	300/1650	23.396	76.604	10.20	21.75	14.436	30.218
350│15	300/2600	30.206	69.794	9.00	17.70	10.742	32.352
375│15	300/2850	25.520	74.480	8.55	14.25	13.504	38.176
250│15	600/1600	22.286	77.71	15.60	22.20	12.895	27.019
300│15	600/2200	21.406	78.594	13.00	27.15	9.578	28.866
350│15	600/3150	26.840	73.160	9.00	17.70	11.201	35.259
375│15	600/3500	22.440	77.560	6.66	14.25	18.073	38.577
300│0	600/2200	17.380	82.620	13.36	29.25	10.674	29.336
300│30	600/2200	20.020	79.980	10.50	25.20	17.369	26.911
300│60	600/2200	19.360	80.640	8.10	23.55	17.541	31.449

**Table 2:** Reaction constant rates obtained for ultrasound-assisted liquefaction of cork (Adapted from [78]).

Ultrasounds Amplitude %	Rate Constant (k) (×10^−2^)	Correlation Coefficient (R2)
0 (Conventional)	1.20	0.83
60	1.59	0.95
80	2.47	0.87
100	5.03	0.98

Ultrasounds Amplitude %

Rate Constant (k)

(×10^−2^)

Correlation Coefficient (R2)

**Table 3:** Dynamic mechanical and thermal properties of polyurethane resins [94].

Sample	T_g_ (°C)	E’ (MPa) at 25 °C
Ag_PU	−34.9	839.9
AG_1.2PU	−42.3	651.6
OG_PU	−52.3	505.1
OG_1.2PU	−36.1	671.1
AGP_PU	−43.5	609.8
AGP_1.2PU	−47.1	339.9
OGP_PU	−48.5	665.3
OGP_1.2PU	−49.7	351.8
AP_PU	−51.8	114.9
AP_1.2PU	−47.9	62.4
OP_PU	−45.2	358.4
OP_1.2PU	−54.6	232.6

**Table 4:** The relative molecular weights of the liquefied products at different liquefaction times [95].

Sample	Number-Average Molecular Weight (*M_n_*)	Weight-Average Molecular Weight (*M_w_*)	Peak Molecular Weight (*M_p_*)	Polydispersity
20 min	144	149	145	1.04
50 min	232	241	233	1.04
80 min	209	218	210	1.04
110 min	237	246	238	1.04
140 min	219	228	220	1.04

**Table 5:** Foam formulations for the liquefied biomass-based polyols [97].

	Ingredients	Parts by Weight
A liquid	Biomass-based polyol (12.5% biomass con.)	100
	Catalyst (DBTL)	1 to 1.5
	Surfactant (SH-193)	2 to 2.5
	Blowing agent (water, including water from Neutralization with NaOH solution)	2 to 4.25
	Additives (PEG 400)	15
B liquid	MDI	100 to 240
(Isocyanate index)		80 to 120

**Table 6:** Cell diameter, physicomechanical properties of the bio-foams, and cellulose nanocrystals reinforced bio-foams [99].

Sample ID	Cell Diameter (mm)	Density (kg∙m^−3^)	Thermal Conductivity (mW∙m^−1^∙K^−1^)	Young’s Modulus (kPa)	Compressive Stress (δ 10% kPa)
PU0	4.1±1.1	26.8 ± 1.6	40.4 ± 1.7	272.1 ± 67.6	25.3 ± 2.5
PU5	3.9 ± 0.8	26.1 ± 1.0	39.9 ± 1.8	258.3 ± 65.1	24.8 ± 3.5
PU10	3.3 ± 0.7	20.7 ± 1.3	38.1 ± 1.9	151.4 ± 15.6	12.1 ± 1.4
PU15	0.5 ± 0.1	21.0 ± 1.5	38.0 ± 1.2	500.3 ± 81.7	35.7 ± 4.8
PU20	0.6 ± 0.3	22.1 ± 2.1	37.2 ± 1.7	592.1 ± 81.1	37.3 ± 4.8
PU25	1.9 ± 0.4	23.3 ± 2.3	39.7 ± 1.9	197.8 ± 36.8	14.3 ± 2.1
PU30	2.4 ± 0.5	23.1 ± 3.0	39.6 ± 0.8	171.7 ± 43.9	12.0 ± 1.7
PU35	2.8 ± 0.8	24.2 ± 2.6	39.8 ± 1.9	172.7 ± 46.4	11.6 ± 1.8
PU401	3.1 ± 0.7	26.6 ± 2.0	41.1 ± 1.4	327.2 ± 46.4	17.8 ± 1.7
PU402	0.9 ± 0.3	24.7 ± 2.6	36.1 ± 1.9	459.2 ± 28.2	22.0 ± 1.3
PU403	0.3 ± 0.1	24.8 ± 1.1	35.8 ± 1.9	681.4 ± 132.9	22.3 ± 2.9
PU404	0.2 ± 0.1	24.2 ± 0.7	34.4 ± 0.8	1186.6 ± 163.3	28.7 ± 4.5
PU405	0.2 ± 0.1	23.3 ± 2.3	34.4 ± 1.3	976.6 ± 137.4	26.6 ± 3.2
PU406	0.2 ± 0.1	20.6 ± 0.8	34.0 ± 1.1	857.8 ± 114.5	25.6 ± 3.5
